# Characterization in respect to degradation of titanium‐coated polypropylene surgical mesh explanted from humans

**DOI:** 10.1002/jbm.b.35221

**Published:** 2023-01-07

**Authors:** Nicholas T. H. Farr, Bernd Klosterhalfen, Günter K. Noé

**Affiliations:** ^1^ Department of Materials Science and Engineering University of Sheffield Sheffield UK; ^2^ Insigneo Institute for in silico Medicine Sheffield UK; ^3^ Institute for Pathology at the Düren Hospital Düren Germany; ^4^ Department of Obstetrics and Gynecology Rheinlandclinics Dormagen University of Witten Herdecke Dormagen Germany

**Keywords:** degradation, materials characterization, oxidation, pelvic organ prolapse, polypropylene mesh, stress urinary incontinence, titanium coating, transvaginal mesh, titanium mesh

## Abstract

Titanium‐coated polypropylene (Ti‐PP) mesh was introduced in 2002 as a surgical mesh for the treatment of hernias and shortly after for pelvic floor surgery, with the aim of improving biocompatibility when compared to non‐titanised/regular PP mesh implants. The application of a titanium coating could also be beneficial to address concerns regarding the exposure of PP in an in vivo environment. Many studies have shown that PP, although it is widely accepted as a stable polymer, is subject to oxidation and degradation, such degradation affects the mechanical behavior, that is, the stiffness and tensile strength of PP mesh. Despite the wide clinical use of Ti‐PP surgical meshes, no study has yet investigated the residual material properties post clinical deployment and subsequent explantation. In this study, two explanted Ti‐PP mesh samples each having different incorporation durations from two patients were examined. Material analysis conducted within this study includes the following techniques: attenuated total reflectance‐Fourier transform infrared spectroscopy (ATR‐FTIR), Raman spectroscopy, low voltage – scanning electron microscopy (LV‐SEM), backscattered electron (BSE) imaging, energy dispersive X‐ray spectroscopy (EDS) and secondary election hyperspectral imaging (SEHI). The hypothesis of this study is that the Ti coating successfully shields the PP mesh from oxidative stress in vivo and thus protects it from degradation. The results of this analysis show for the first time evidence of bulk oxidation, surface degradation, and environmental stress cracking on explanted Ti‐PP meshes.

## 1 INTRODUCTION

Surgical mesh made of polypropylene (PP) has been used since the 1950 s to repair abdominal wall hernias such as incisional and inguinal hernias.^1^, ^2^ In the 1970 s, gynecologists started using meshes for pelvic organ prolapse (POP) and about 20 years later, gynecologists began using PP meshes for treatment of stress urinary incontinence (SUI).^3^ At around the same time, surgical meshes were first used in transvaginal repair of POP. Due to the poor biostability of PP, PP mesh is nowadays always combined with various additives like antioxidants.^4^ However, explanted PP mesh was frequently reported to show surface alterations such as cracks and flaking.^5^, ^6^ Degradation of PP mesh is considered to be the result of the synergistic effects of corrosive chemicals such as reactive oxygen species (ROS) produced by inflammatory cells and tensile stress in the mesh fibers in vivo.^7^, ^8^ Degraded PP mesh has reduced tensile strength and elastic modulus, the altered morphology of the mesh surface also affects the interaction with the immune cells.^9^, ^10^ Therefore, the concern about the long‐term safety of PP mesh implants is growing.^11^, ^12^

Titanium (Ti) is widely used as a material for implants such as dental and ossicular implants or knee and hip joint prostheses because of its high corrosion resistance and tissue compatibility.^13^, ^14^ Since titanium cannot be directly used as a mesh material due to its lack of flexibility and elasticity, a process of coating PP mesh fibers with Ti was developed with the intention of improving biocompatibility. Ti‐coated PP (Ti‐PP) mesh was introduced to the market in 2002, initially for the treatment of hernias and later for pelvic floor surgery. It is claimed that Ti‐PP mesh has a homogeneous Ti coating that is covalently bonded to the PP mesh fibers.^15^, ^16^ The hypothesis of this study is that the Ti coating successfully shields the PP mesh from oxidative stress in vivo and thus protects the mesh from degradation. To the authors' knowledge, no study on the degradation of Ti‐PP mesh post implantation has been carried out so far. However, this is warranted for implants intended for a long incorporation duration, ranging from 10 to 50 years depending on the age of the patient, among other things.

This study therefore evaluates the material properties of two explanted Ti‐PP mesh samples from patients who have undergone pelvic floor surgery. Both Ti‐PP meshes were explanted due to recurrence after different incorporation durations in the body. Following recommendations of the ISO 10993‐18 standard, a range of analysis techniques were applied to study the explanted meshes at the multiscale. Bulk oxidation was assessed by attenuated total reflectance – Fourier transform infrared spectroscopy (ATR‐FTIR), surface morphology degradation by low voltage – scanning electron microscopy (LV‐SEM) in combination with backscattered electron (BSE) imaging and an assessment of surface/subsurface chemistry was performed using energy dispersive X‐ray spectroscopy (EDS), Raman spectroscopy and secondary election hyperspectral imaging (SEHI). The aim of the study is to investigate whether Ti coating prevents the degradation of PP mesh in vivo. In addition, the data collected in this study was compared with PP data available from other studies.^5^, ^17^, ^18^, ^19^

## 2 MATERIALS AND METHODS

### 2.1 Materials

Two Ti‐PP meshes which were implanted in female human pelvic floors and then explanted after approximately 6 (short‐term; ST) and 24 (long‐term; LT) months due to recurrence at the University of Witten Herdecke, Rheinlandclinics Dormagen, Germany, were examined.

**FIGURE 1:**
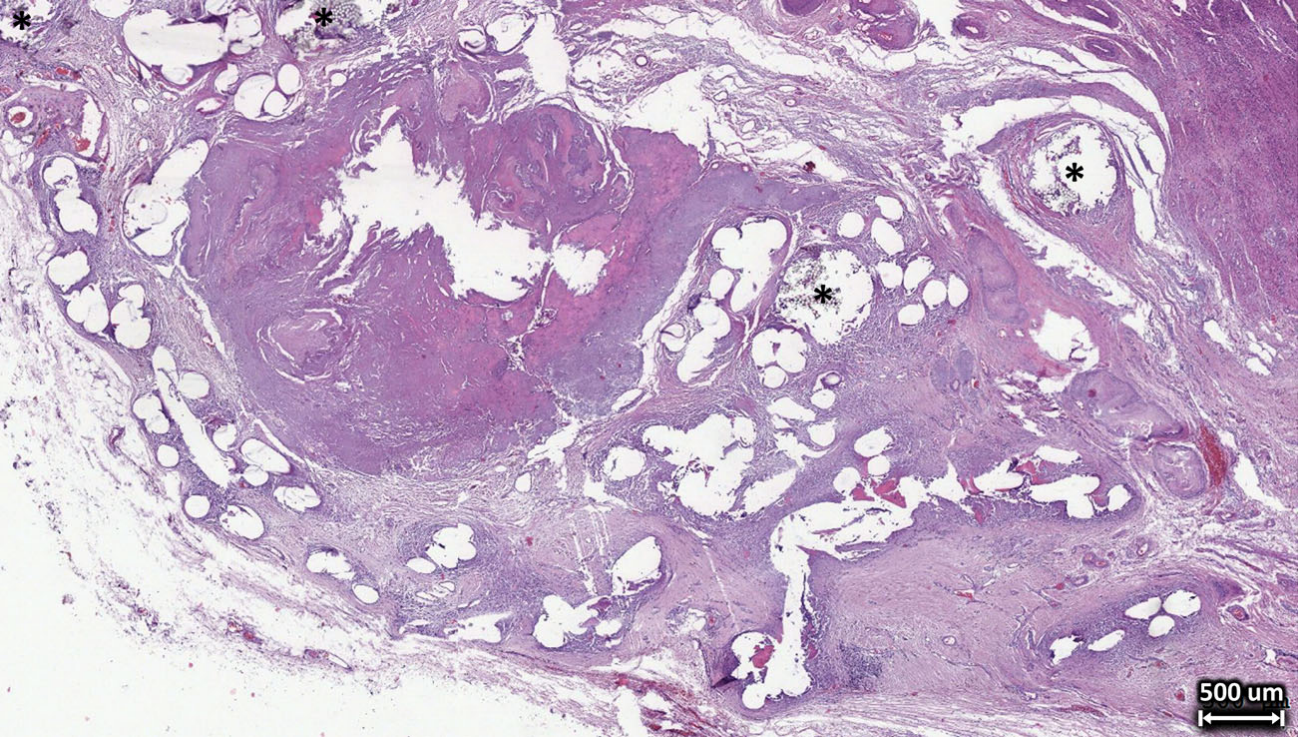
Tissue section of the long‐term (24 months) implanted titanium‐coated polypropylene (Ti‐PP) mesh stained with hematoxylin and eosin showing a persistent strong foreign body reaction with severe fibrosis and marked mesh shrinkage with visible wrinkling. Note that multifilament suture material is also visible in some areas (black asterisks). The remaining round, elliptical or elongated gaps in the tissue section are from mesh fibers

**FIGURE 2:**
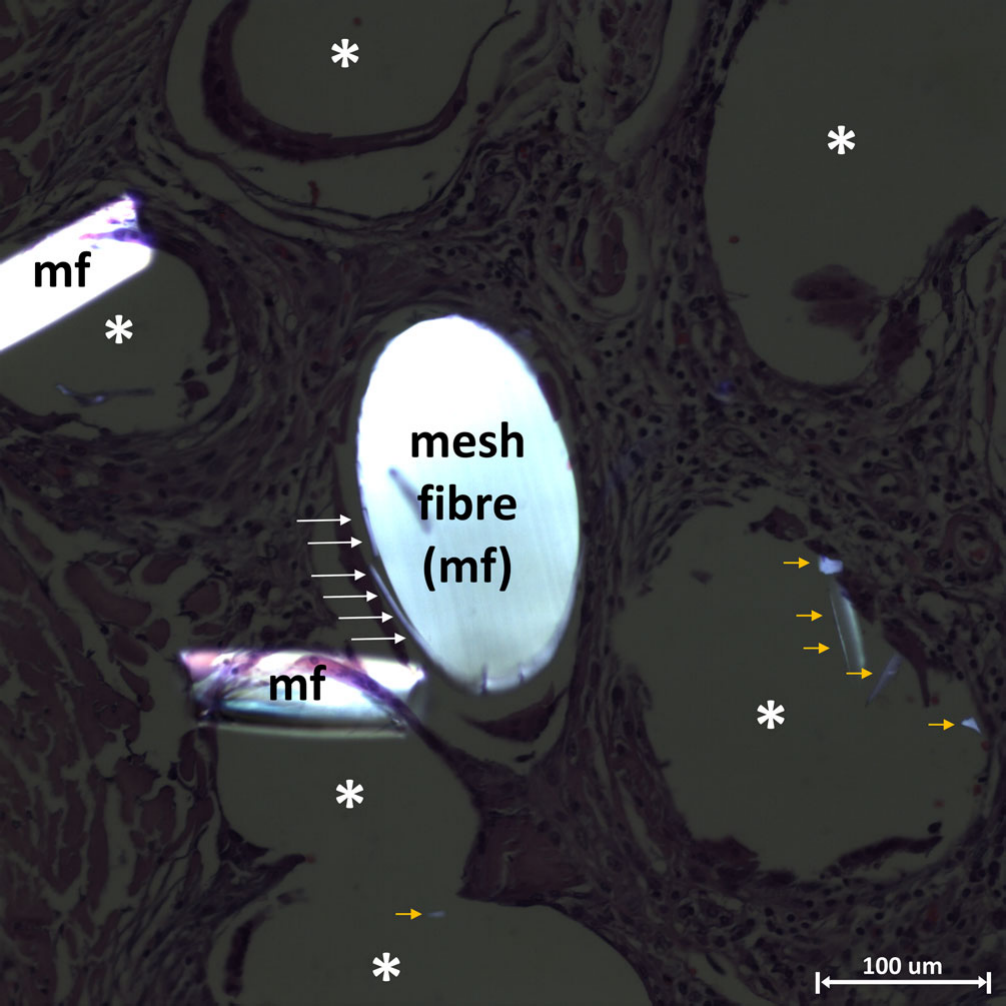
Brightfield image taken with polarized light to test whether synthetic material is still present and to investigate degradation. The image is from the long‐term implanted titanium‐coated polypropylene (Ti‐PP) mesh. In the center, a filament can be seen that has been cut perpendicular to its length, with a laterally visible peeling or flaking of the material due to degradation (white long arrows). Nearby are two coiled fibers. There are also several birefringent synthetic fragments visible (orange short arrows); mf, mesh fiber; white asterisks, mesh fiber gaps

According to the pathological reports from the Institute for Pathology at the Düren Hospital, Germany, tissue samples of both explants showed a persistent strong foreign body reaction with severe fibrosis and marked mesh shrinkage with visible wrinkling of the mesh (Figure 1). There was no low‐ or high‐grade infection and no altered nerve structures. Both explants showed visible mesh degradation when examined with polarized light (Figure 2). Consistent with the incorporation times, ST mesh showed slight degradation, while LT mesh showed advanced degradation.

Prior to materials characterization, the explanted Ti‐PP mesh samples were thoroughly cleaned by a previously established cleaning process to remove any biological tissue adhering to the mesh surface.^19^ Briefly, samples were treated with an alkaline solution (pH 8.5–9.5) containing protease (Alcalase® 2.5L) at 58°C for 12 h. Common salt, tenside SUPRALAN UF (Zschimmer & Schwarz, GER) and sodium carbonate were added to the solution to regulate the pH value. The protease Alcalase® is a serine endopeptidase that consists primarily of subtilisin A, which is suitable for the hydrolysis of proteins and harmless to synthetic mesh materials, that is, the cleaning process does not affect the mesh fibers. After an incubation period of 12 h, the meshes were rinsed with water. For use of comparison, a Prolene® Mesh (Ethicon) was selected as a control. The Prolene® Mesh (Pristine PP) was washed with deionized water prior to analysis.

### 2.2 Attenuated total reflectance – Fourier‐transform infrared spectroscopy

Infrared spectra were obtained for both short term (ST) and long term (LT) implanted Ti‐PP mesh samples as well as a pristine PP mesh sample with a NICOLET 380 FTIR spectrometer (ThermoFisher Scientific, USA). Purged with dry air before spectra collection in the range from 500 to 4000 cm^−1^ averaging 32 scans and a resolution of 4 cm^−1^. The samples were analyzed in their solid state form using an attenuated total reflection accessory with a Golden Gate® diamond crystal (Specac, UK).

### 2.3 Energy dispersive X‐ray spectroscopy

FEI Helios Nanolab G3 (FEI Company, USA) SEM equipped with an Energy Dispersive X‐ray Spectroscopy (EDS) detector (Oxford Instruments, UK) was used to capture EDS spectra. EDS spectra were taken from the center of each Ti‐PP mesh filament to mitigate any effects associated with fiber orientation. The spectra were obtained with a 10 keV accelerating voltage using a 13 nA probe current at a working distance of 5 mm. Data analysis was automated by the application of Aztec EDS analysis software (Oxford Instruments, UK).

### 2.4 Low voltage‐SEM imaging

FEI Helios Nanolab G3 (FEI Company, USA) and Helios G4 DualBeam (ThermoFisher Scientific, USA) microscopes were employed for surface morphology observations of both explanted Ti‐PP mesh samples. In contrast to common SEM analysis practice, samples were not previously treated with a conductive coating by deposition. This approach was selected to aid in the visualization of the Ti coating present on the Ti‐PP meshes. An accelerating voltage of 1–2 keV at typical chamber vacuum pressures in the range of 10^−6^ mbar and a working distance of 4 mm were chosen to avoid sample damage through surface charging. An Everhart–Thornley Detector (ETD) was selected for low magnification of SE images and a Through Lens Detector (TLD) for high magnification SE images.

### 2.5 Backscattered electron imaging

BSE Images were obtained using a concentric backscatter (CBS) detector housed within a FEI Nova Nano 450 SEM (FEI Company, USA). BSE collection was performed at 2 keV, with typical chamber vacuum pressures in the range of 10^−6^ mbar at a working distance of 5 mm.

### 2.6 Secondary electron hyperspectral

In previous studies, the process of SEHI data acquisition has been described in great detail.^20^, ^21^ Briefly, SEHI generation in this study was performed using the Helios Nanolab G3 and Helios G4 microscopes by applying consistent operating conditions of 1 keV (monochromated) and 50 pA immersion mode (mode II/UHR). These microscopes are capable of providing ultrahigh resolution images at voltages <1 keV. To ensure that images were taken of the true material surface, no conductive coating was applied to the samples in contrast to typical SEM analysis practice. At the time of analysis, a typical vacuum pressure of ~10^−6^ mbar was maintained at a working distance of 4 mm. The collection of SEHI of different energy ranges was enabled through the adjustment of the mirror electrode voltage (MV) together with a tube bias setting of 150 V. Stepping the MV in a range of −15 to 15 V was achieved through the use of an automatic iFast collection recipe.^22^ Every image was acquired at a frame interval of 0.5 s and an MV step size of 0.5 V, corresponding to an electron energy step size of about 0.2 eV. Image processing was done with Fiji ImageJ software (ImageJ2, open‐source).

### 2.7 Raman spectroscopy

Raman spectroscopy (Renishaw inVia micro‐Raman) was employed to analyze the chemical structure of Pristine PP mesh along with ST and LT Ti‐PP mesh explants. Raman spectra were collected from longitudinal fibers located distal from mesh knot sites. A 50× objective was selected with 10 s exposure. The laser power was set at 3 Mw with a 1 μm spot size. Peltier‐cooled multichannel CCD detector was used for data recording with a 2400 lines/mm diffraction grating at a slit opening of 65 μm and a spectral resolution of in the order of 1 cm^−1^. For data analysis no smoothing was applied with baseline subtraction performed using OriginLab (OriginLab Corporation, USA) software.

## 3 RESULTS AND DISCUSSION

### 3.1 Quantification of bulk oxidation by ATR‐FTIR spectroscopy

**FIGURE 3:**
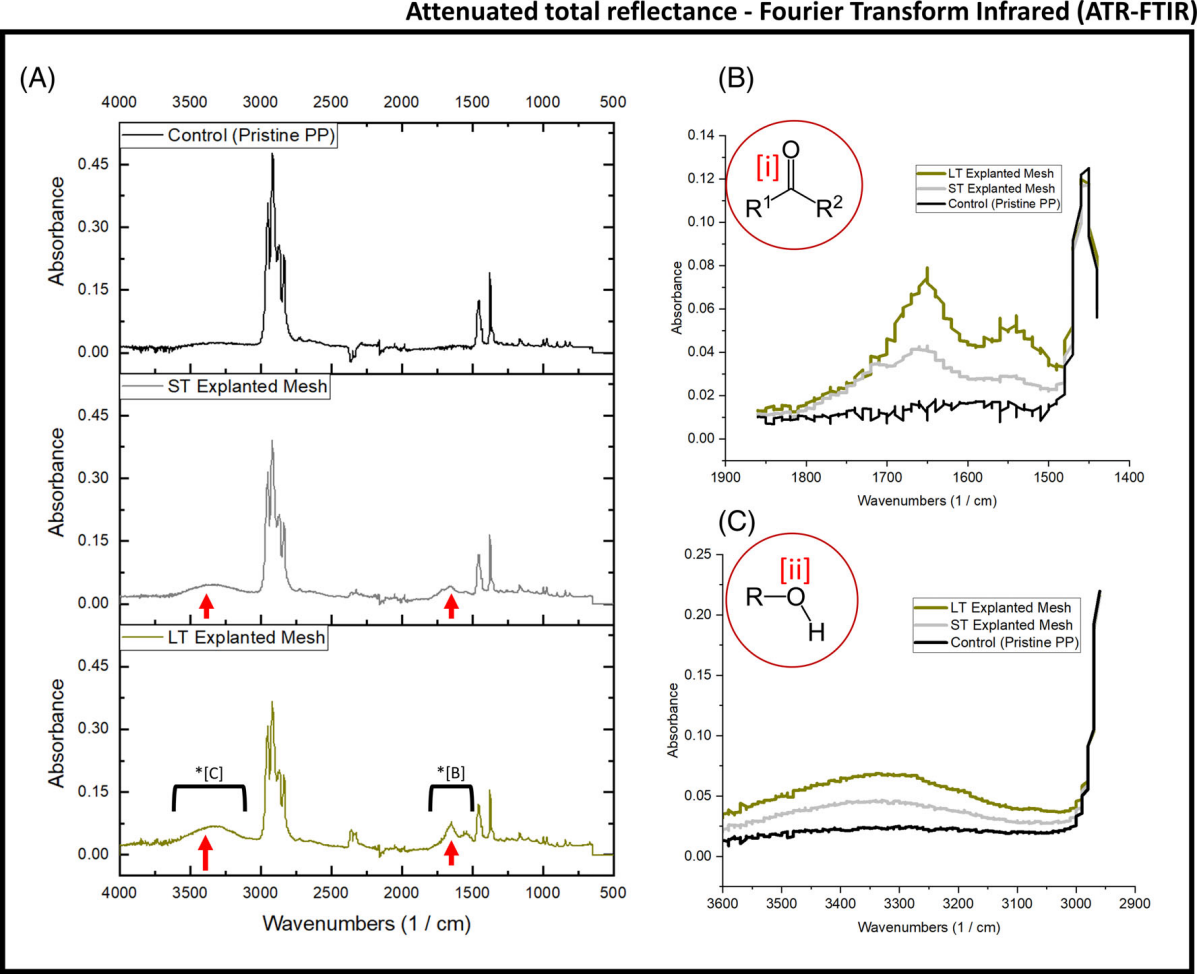
(A) Attenuated total reflectance‐Fourier transform infrared spectroscopy (ATR‐FTIR) spectra collected from pristine PP mesh as well as short‐term (ST) and long‐term (LT) explanted titanium‐coated polypropylene (Ti‐PP) meshes (all meshes described in 2.1). Both spectra of the explanted meshes show a carbonyl (—C=O) and hydroxyl (—OH) peak at 1600 cm^−1^ and 3400 cm^−1^, respectively, which increase compared to the control sample. (B–C) show the superimposed localized spectra; (B) —OH peaks integrated from 3600 to 3000 cm − 1 and (C) —C=O peaks integrated from 1850 to 1450 cm^−1^. (i, ii) show the oxidation products of carbonyl and hydroxyl groups

To obtain a measure of the bulk oxidation for both the short‐term (ST) and long‐term (LT) explanted Ti‐PP mesh samples, ATR‐FTIR was performed. FTIR is recommended for determining the chemical structure of synthetic polymers used in medical devices according to ISO 10993‐18:2020 standard. Figure 3A presents the FTIR spectra obtained from a pristine PP mesh sample (Prolene® Mesh, Ethicon) and both explanted Ti‐PP meshes. FTIR spectra of both explanted meshes show the appearance of pronounced carbonyl (—C=O; expected range: 1750–1500 cm^−1^) and hydroxyl (—OH; expected range 3600–3000 cm^−1^) groups compared to the pristine PP sample, which served as control. Noteworthy, LT mesh showed a significant increase in both —C=O and —OH groups compared to that of the ST mesh, which in turn showed an increase compared to the control (Figure 3B,C). Consequently, these results demonstrate that despite the Ti coating, the bulk PP increasingly oxidizes over time in vivo, which is consistent with previous studies on uncoated PP meshes.^5^, ^17^, ^18^, ^19^ Studies have long documented the oxidation of PP,^23^, ^24^ which includes the formation of a hydroperoxide (—ROOH) prior to chain scission, with subsequent formation of aldehydes, ketones, and carboxylic acids, with accompanying chain cleavage.^4^, ^25^ This oxidation mechanism has been proposed for PP degradation in vivo^17^; with the formation of hydroperoxide (—ROOH) prior to chain scission and subsequent formation of a carbonyl (—C=O) end group.^4^, ^17^, ^26^, ^27^ This oxidation pathway is consistent with the data obtained in this study. The consequences of PP oxidation on fiber properties have been considered and studied extensively, including fiber cracking/crazing, flaking, molecular alterations, loss of elasticity, and embrittlement.^5^, ^17^, ^27^ The evidence shown here for bulk oxidation of Ti‐PP mesh makes it clear that Ti coating does not prevent PP from degradation in vivo.

### 3.2 Evaluation of surface morphology by SEM, BSE and SE imaging

**FIGURE 4:**
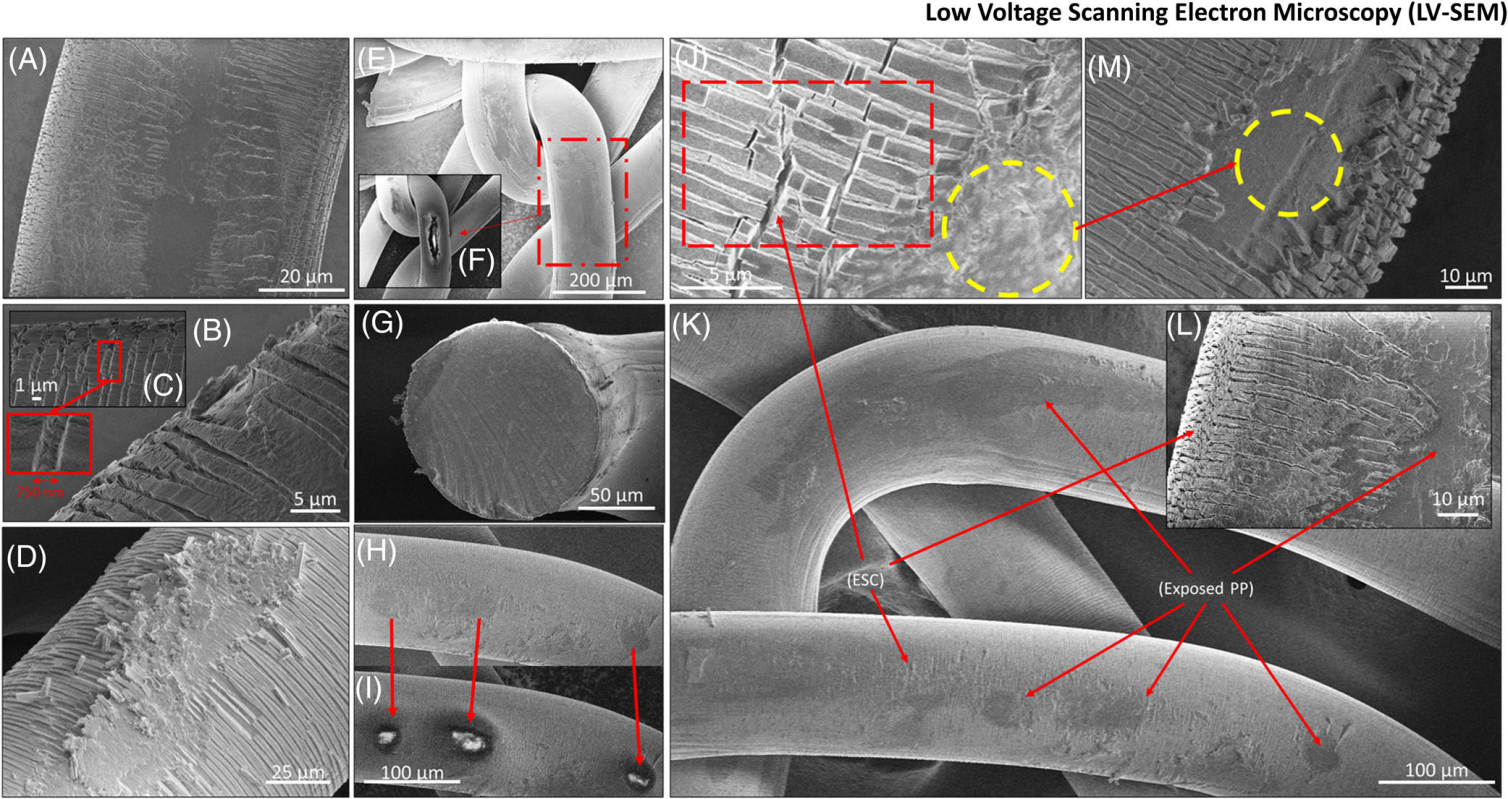
Collection of scanning electron microscopy (SEM) images of the short‐term (ST) and long‐term (LT) implanted titanium‐coated polypropylene (Ti‐PP) mesh. (A) ETD image captured to visualize the surface morphology of ST mesh. Surface aberrations and areas with environmental surface cracking (ESC) are present. (B, C) Both images are secondary election (SE) images captured from the ST mesh with TLD detector in immersion mode. The images show ESC at a higher resolution. Notably, crazing, that is, internal fracturing without a change of the surface texture, can be seen in cracks in the Ti coating, which in this example measured a width of 750 nm. (D) This image of the LT mesh was acquired with the same high‐resolution TLD setup. Comparison of images (B) and (D) shows that the captured LT mesh region has greater surface fragmentation, which is consistent with the results from the attenuated total reflectance‐Fourier transform infrared spectroscopy (ATR‐FTIR) analysis. (E, F) presents another high‐resolution TLD image of the ST mesh. In this image, there are regions (one highlighted) that appear to have different surface morphology than the rest. These regions are surrounded by areas of cracked Ti coating and appear to be exposed PP material. In image (F), the accelerating voltage was increased from 1 to 2 keV. The reason for this is that by increasing the voltage, the Ti‐coated regions, which conduct well, are not charged as much as the exposed PP. Consequently, the regions with altered surface morphology represent exposed PP. (G) shows an ETD‐SE image of a mesh fiber from the ST mesh after cryo‐fracturing to reveal its cross‐section. The fracturing has clearly left marks (from top left to bottom right) corresponding to the fracture motion. The image shows the outer edge of the Ti coating and PP bark as well as some loosely adhering particles. Figure S1 allows for Ti coating thickness comparison between an explanted fiber and a previously published image of cross‐section of a Ti mesh prior to implantation. (H, I) are similar images to (E, F), but from the LT mesh. Interestingly, exposed PP regions for the LT mesh were also located further away from areas subject to high mechanical stress, such as mesh knots, unlike for the ST mesh. This shows that PP is not only exposed in regions of high mechanical stress, but that there may be several overlapping mechanisms leading to oxidation and surface alterations. For example, myeloperoxidase secreted by macrophages, among others, is known to generate reactive oxidized species that have been suggested to play a role in PP mesh surface alterations and changes in mechanical behavior. (J, M) show high‐resolution TLD‐SE images from the LT and ST mesh, respectively. Of note, within this image is the highly fragmented, longitudinal cracking within the coating (J) situated next to another exposed region. Comparing (J) with (M) it is notable to visualize differences in cracking patterns, common between the two meshes. (K, L) show the extent of ESC and exposed PP regions at the LT mesh at different scales

In order to evaluate the surface morphology of the two explanted Ti‐PP meshes, a series of SEM images were acquired (a methodology recommended by ISO 10993‐18). The analysis was performed on uncoated samples, that is, no additional conductive coating was applied to directly visualize the surface morphology of the Ti‐PP meshes. To mitigate the impact of sample charging and to generate highly surface sensitive images a low voltage‐SEM (1–2 keV) imaging was employed with various detector set ups; ETD and TLD in both field free and immersion mode. The resulting images are presented in Figure 4.

From the images in Figure 4, it is apparent that both environmental stress cracking (ESC) and surface degradation has occurred on both explanted Ti‐PP meshes. The Ti coating does not prevent the degradation of PP, which is visible through cracks and flaking of the fiber surface on SEM images. This observation of cracks and flaking is consistent with the results of studies on non‐titanised explanted PP meshes.^5^, ^6^, ^28^, ^29^

The effects of mesh degradation on the biological host response are not yet clear. However, the release of PP particles in the nano‐ to micrometer range irritate cells and stimulates the immune system^9^ or contribute to increase inflammation via surface enlargement and may explain the relatively strong foreign body reaction of non‐titanised PP. In addition, it is known that mesh degradation affects the mechanical behavior, that is, the stiffness and tensile strength.^4^, ^30^, ^31^, ^32^, ^33^, ^34^

When comparing the two explanted Ti‐PP mesh samples, the short‐term (ST) implanted mesh displays less ESC and surface degradation than the long‐term (LT) implanted mesh. It can also be observed that the apparent exposed regions of PP are likely to be found on the surface of the LT mesh, both on straight fibers and in areas of high local mechanical stress, that is, knot sites. It is noticeable that the cracks on the surface of the Ti‐PP mesh fibers generally occur more frequently on the outside of the fibers, which are bent by the mesh's knitting structure. To accommodate the degree of bending, the outer surface of the mesh fiber is stretched while the inner surface is compressed. Among other things, this can explain why the surface morphology of materials that are not stable in vivo changes over time.

**FIGURE 5:**
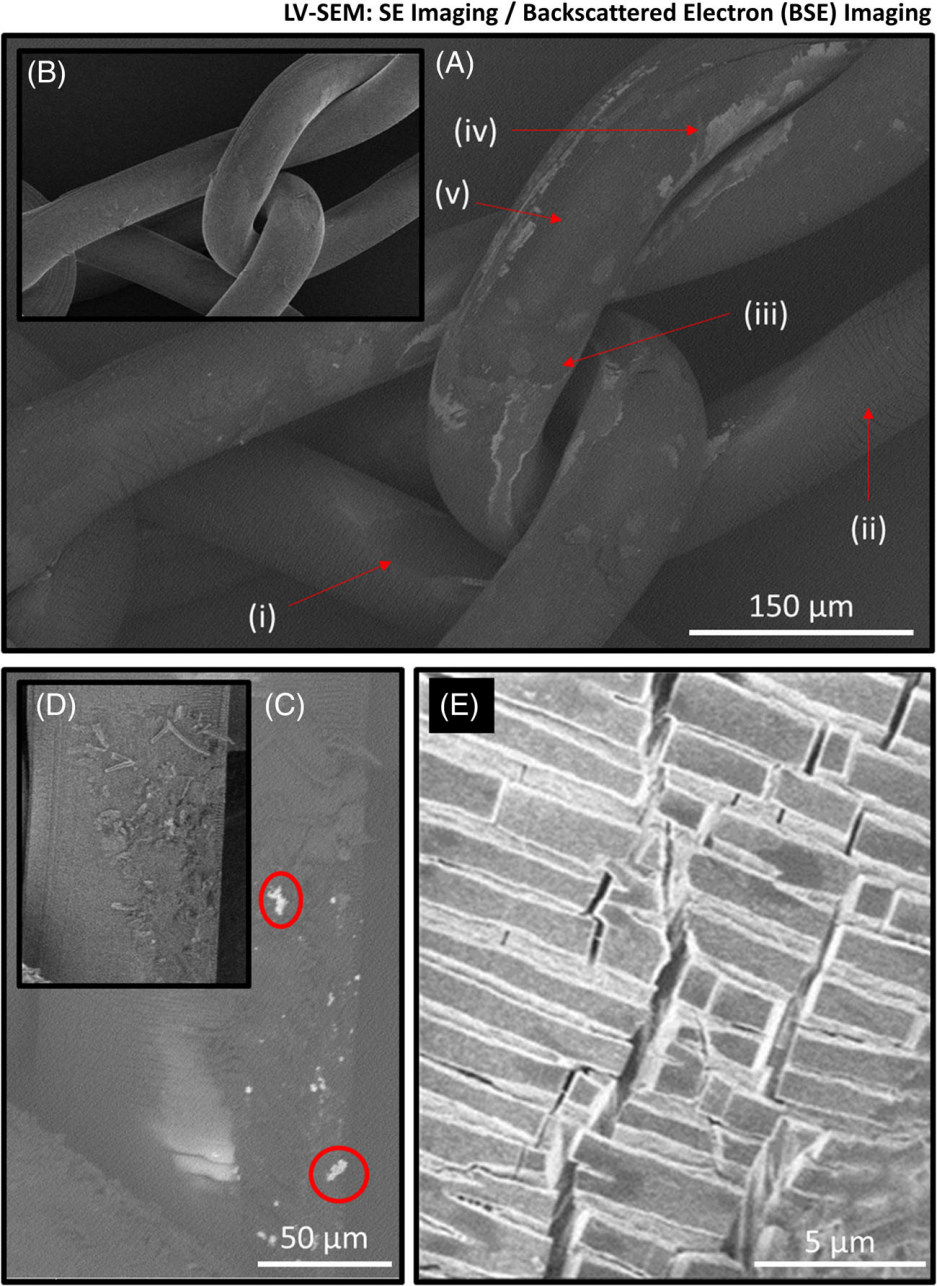
Collection of secondary electron (SE) and back scattered electron (BSE) images of short‐term (ST) and long‐term (LT) implanted titanium‐coated polypropylene (Ti‐PP) mesh. (A) BSE image taken from the same region as the inset SE image in (B) of the ST mesh. In (A), the red arrows (i)–(v) refer to areas of Ti‐PP surface degradation. (C) BSE image taken from LT mesh at the same region as the SE image in (D). The included red circles in (C) highlight structures of high BSE contrast. (E) Close‐up SE image of a mesh fiber of the LT mesh showing the pattern of dense surface cracks orthogonal to the longitudinal direction of the mesh fiber

To better visualize surface degradation, BSE imaging was performed in addition to high‐resolution secondary electron (SE) imaging (Figure 5). SE imaging is a long‐established SEM‐based characterization method whose contrast depends on the atomic number of the material; the larger the atomic number, the more backscattered electrons are detected. Figure 5A,B compare a BSE and SE image of the ST mesh. Figure 5A shows exposed regions around high stress knot sites comparable to the ETD images obtained and presented in Figure 4.

The BSE image in Figure 5A, shows larger contrast variation compared to that of the SE image (Figure 5B). The darkest BSE contrast areas, such as (i), appear in regions previously hypothesized as exposed PP. This image shows that the removal of Ti coating (ii) exposes a lower atomic number material, in this case probably PP. Figure 5A (iii) points to a region of low contrast BSE emission, which is likely exposed PP, surrounded by high‐contrast areas similar to the Ti coating in Figure 5A (ii) and even higher‐contrast regions present mainly at the inner fiber edge (Figure 5A iv) of touching mesh fibers. The chemical composition of these high BSE contrast regions is unknown but a potential explanation for this observation would be degradation to the Ti coating. Within the region in Figure 5A (iii), there is a slightly higher BSE contrast region (Figure 5A v). The contrast in this region is lower than in Figure 5A (ii), so it is unlikely to be related to the Ti coating. It is therefore considered that the results from this region may be associated with surface alterations of the exposed PP.

Similar to Figure 5A–D compare a BSE image with a high‐resolution SE image, respectively. Figure 5D shows fragmented Ti‐PP regions similar to images presented in Figure 4. In Figure 5C a BSE image taken from the same region as Figure 5D shows a collection of high BSE contrast structures. These bright contrast structures range from 1–10 of microns in size and are situated in a highly fragmented Ti‐PP surface region. Figure 5E shows a high‐resolution SE image with some darker markings and cracked and dislocated Ti coating. As to be discussed in more detail, the high‐contrast structures observed in Figure 5C are similar to those of the dislocated Ti coating in Figure 4D,J, and in BSE imaging as Ti surface fragments.

### 3.3 Assessment of surface/sub surface chemistry by EDS, SEHI and Raman spectroscopy

**FIGURE 6:**
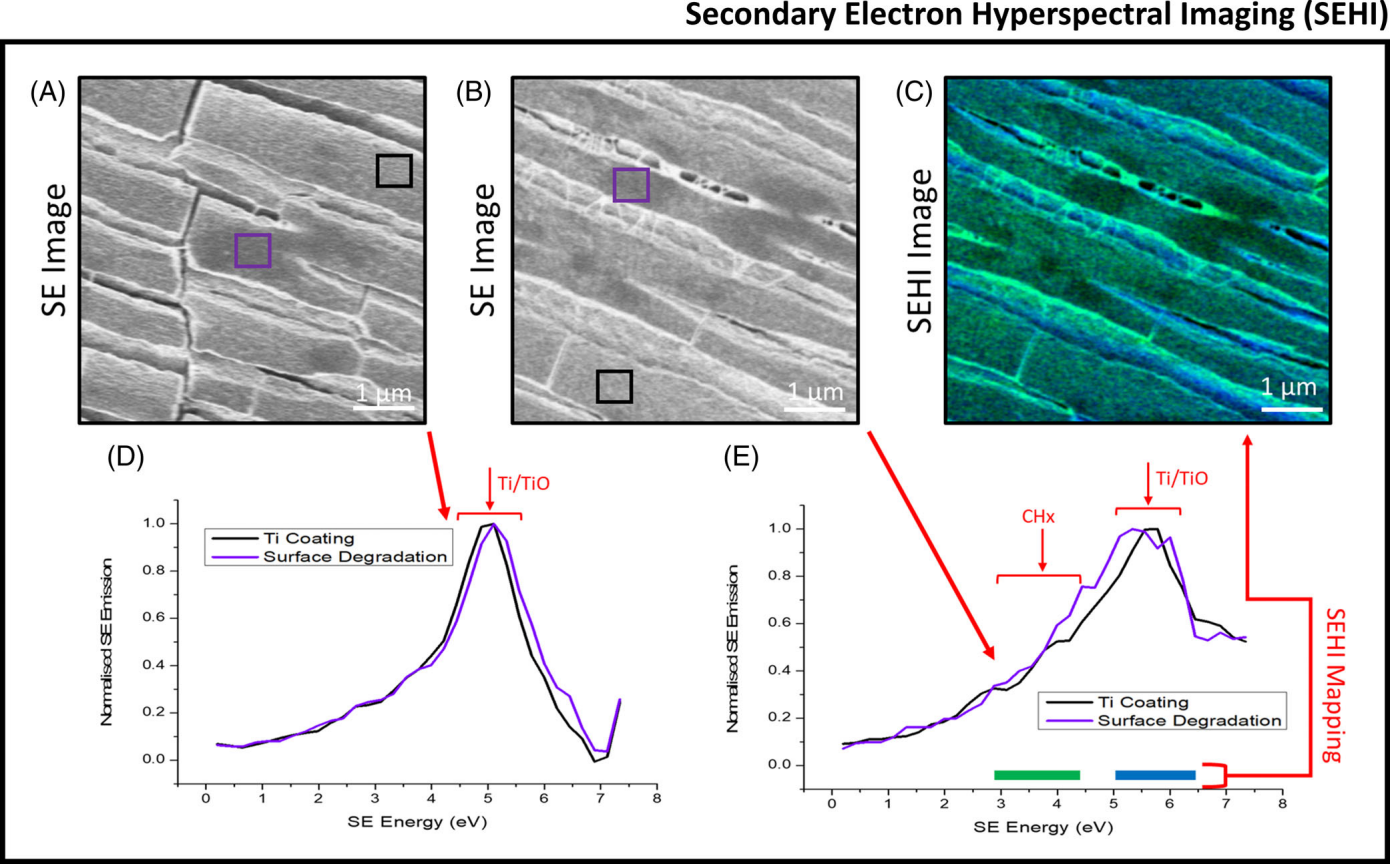
Secondary electron hyperspectral imaging (SEHI) data collected for the short‐term (ST) and long‐term (LT) implanted titanium‐coated polypropylene (Ti‐PP) mesh. (A) and (B) show the respective secondary electron (SE) images of the regions of interest used for the SE spectra collected in (D) and (E). Ti SE peak has previously identified at 4.9–5.3 eV, with TiO found in a peak range of 5.5–6.2 eV. Both SE spectra presented in (D) and (E) show insets highlighting Ti and TiO SE emission peak ranges, with LT mesh exhibiting greater TiO emission compared to that of ST mesh. Image (C) shows an SEHI color map of (B) with color SE ranges obtained as follows: green = 2.9–4.6 eV for CHx and blue = 5.1–6.4 eV for both Ti/TiO

**FIGURE 7:**
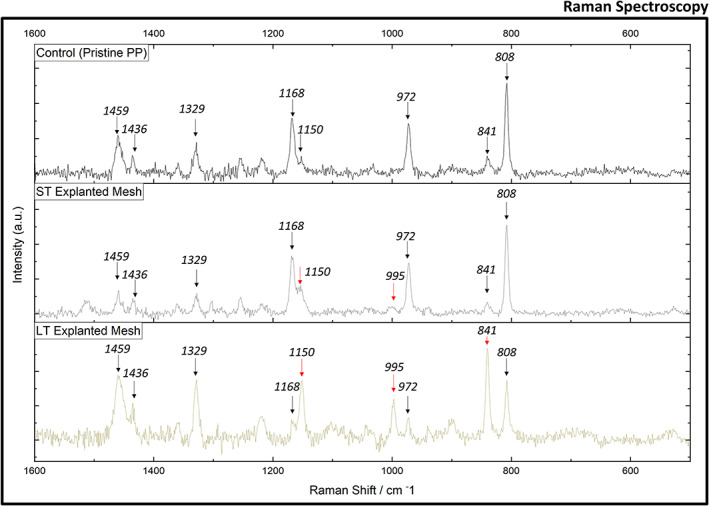
Raman spectra of pristine PP mesh (top) and short‐term (ST, middle) and long‐term (LT, bottom) implanted titanium‐coated polypropylene (Ti‐PP) mesh. Red arrows highlight notable band variances observed in explanted Ti‐PP meshes and compared to the pristine PP mesh

To evaluate the surface chemistry of both explanted Ti‐PP meshes, energy dispersive X‐ray spectroscopy in accordance with the recommendation of ISO 10993‐18:2020 for the identification of metals/alloys in medical devices was performed (Figure S1). However, as the Ti coating is ~30 nm thick, identification is expected to be problematic using EDS, which produces X‐rays in a region of about 2 μm in depth. Therefore, we additionally performed secondary electron hyperspectral imaging (Figure 6) alongside Raman spectroscopy to identify sub‐surface alterations within the chemical structure/crystallinity (Figure 7).

SEHI is a novel, highly surface sensitive technique that has been shown to be able to differentiate bonds on carbonaceous material surfaces^35^, ^36^, ^37^, ^38^, ^39^ and extract information about the work function of metal oxide surfaces.^21^, ^40^ Figure 6A,B show SE images of the regions of interest (ROIs) selected for the SEHI analysis from ST and LT meshes, respectively. Both images show ESC cracking and what appears to be dark surface markings. For the purpose of SEHI analysis, two sub‐ROIs were selected. These are highlighted within both images as purple boxes containing surface markings, previously identified in Figure 4J, and black boxes containing what looks like cleaner Ti coating.

Figure 6D,E show the SE spectra (SES) generated. In previously published SEHI data, a SE Ti peak was identified at 4.9–5.3 eV, with Ti oxides found in a peak range of 5.5–6.2 eV.^20^ Both of these peaks are apparent in all SES spectra collected. We can therefore confirm that Ti is still present on the surface of both explanted Ti‐PP meshes. SES spectra presented also shows emission variances in the peak position related to the presence of Ti/Ti oxides, comparing ST and LT incorporation. This result is interesting; however, it is not within the scope of this study to speculate on surface alterations to the Ti/TiO_2_ coatings.

For both ST mesh and LT mesh, the purple box with surface markings contains a greater Ti oxide SE peak emission than the black box with visually less degraded coating. This result suggests that the markings on the Ti coating could be regions of highly localized surface oxidation. Figure 6E also highlights an additional SE peak range of interest being that of CH vibrations (CHx). CHx has previously been identified to show characteristic SE spectra within the range of 2.9–4.3 eV.^35^, ^36^, ^41^ The CHx emission is most abundant in LT mesh compared to that of ST mesh. One of the limitations of SES is that SE peaks can often show overlapping chemical related emissions. This is common for higher energy emissions (4–6 eV), which are sensitive not only to Ti oxides but also to peaks associated with oxygen functionalities in organic materials. To overcome this limitation and to better understand the composition of the dark surface markings, a SEHI color map was generated for the analysis of the LT mesh (Figure 6C). The SEHI map shows carbon residuals across the Ti coating. Of note is that the carbon is not localized to the dark surface markings. Also, of interest is what appears to be string like structures between the Ti coating located at the edges of the surface cracks. These structures also show strong CHx emission. It is considered that this emission could be related to alkyl groups present within PP or a form of amorphous carbon surface residue.

**FIGURE 8:**
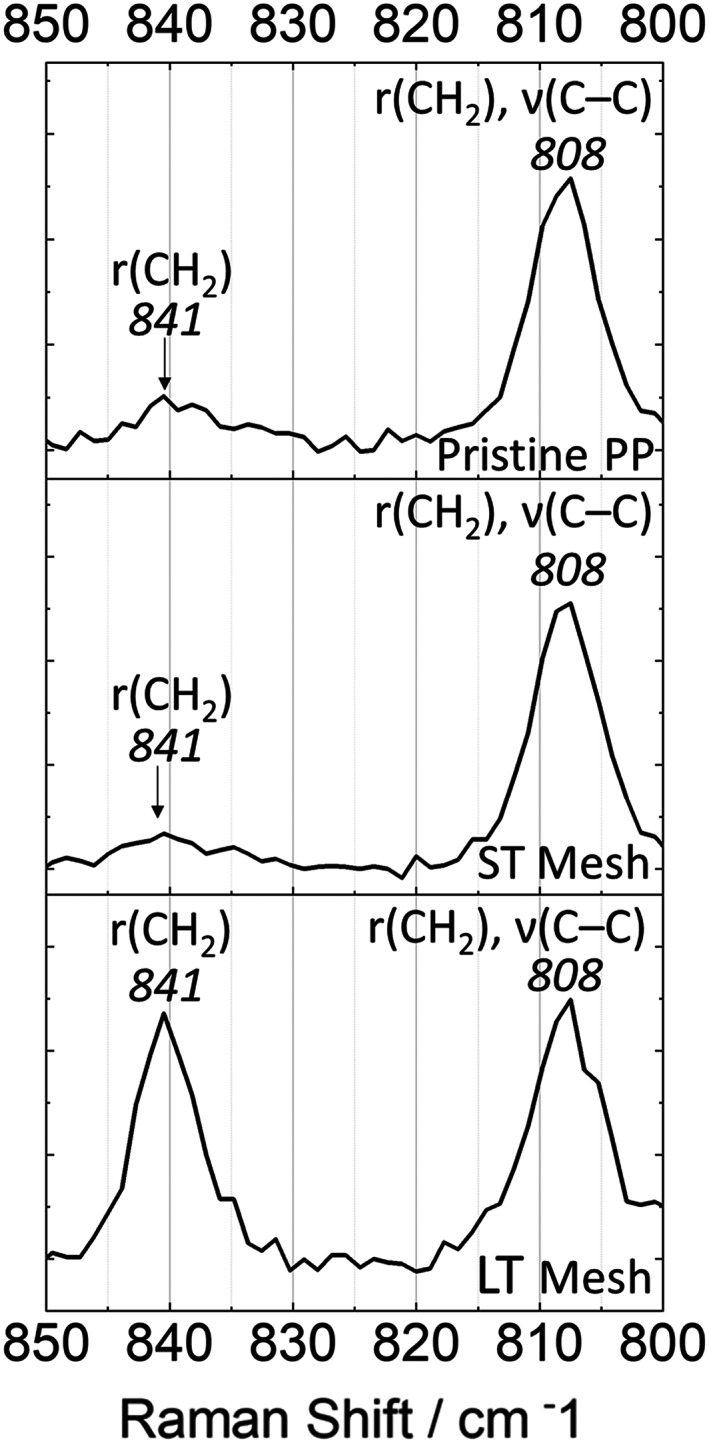
Raman spectra collected between 800–850 cm^−1^ of pristine PP mesh and short‐term (ST) and long‐term (LT) explanted titanium‐coated polypropylene mesh

Raman spectroscopy was performed to identify changes within the structural units of PP backbone structures. Raman spectroscopy is recommended in ISO 10993‐18:2020 for the determination of the constituent structure of synthetic polymers. Figure 7 shows the Raman spectra of pristine PP mesh and ST and LT Ti‐PP meshes recorded in the range 400–1600 cm^−1^. Table 1 contains the vibrational assignment for Raman bands of PP, which has been published in detail.^42^ The spectrum obtained for the pristine PP mesh is consistent with previously published literature.^43^, ^44^ Raman bands obtained from PP within the 1000–1300 cm^−1^ region are not easily assigned as these may be due to various aromatic stretching modes or C—O stretches of the ester functionalities,^45^ which cannot be confirmed without more detailed analysis. It has been previously established that the 808, 841, 972, 995, 1168 cm^−1^ bands are related with the helical chain structure.^42^ Variations within these band ratios, when viewed in terms of relative intensity normalized to PP reference band 972 cm^−1^, reveal a notable helical structural change between the two explanted Ti‐PP meshes and in comparison to the pristine PP mesh. One example of this variation can be seen when examining the band ratio of 808 and 840 cm^−1^ (Figure 8). Both bands have been shown to relate to conformational states of helical chains and have been used previously to estimate the degree of crystallinity in isotactic polypropylene.^43^, ^44^ 808 cm^−1^ is associated with helical chains within crystals (r[CH3] and backbone stretching ν[C—C]), with 840 cm^−1^ assigned to shorter chains in helical conformation.^43^, ^46^ The increase in the intensity ratio I840/I808 has been considered in relation to helical chain disruption/isomeric defects.^47^, ^48^ A loss of C—C coupling results in greater emission from side‐group mode r(CH3), expressed as a shift of the Raman band from 808 to 840 cm^−1^.^43^ When comparing the intensity ratios of I840/I808, it is clear that the LT Ti‐PP mesh expresses greater helical chain defects compared to both the ST Ti‐PP mesh and the pristine PP mesh. A more detailed study, including the analysis of high stress knot sites, is required to generate the required data for better understanding of the mechanisms promoting the observed structural defects.

**TABLE 1:** Vibrational assignment of Raman bands for PP.

Band (cm^−1^)	Vibrational assignment
808	r(CH_2_), ν(C—C)
841	r(CH_2_)
972	r(CH_3_), ν(C—C)
995	r(CH_3_)
1150	ν(C—C), δ(CH)
1168	ν(C—C), r(CH_3_), w(C—C)
1329	t(CH_2_)
1436	δ(CH_2_)
1459	δ(CH_2_)

Abbreviations: r, rocking; t, twisting; w, wagging; δ, bending; ν, stretching.

The direct PP oxidation pathway via the process of chain scission is capable of fragmenting molecular chains. However, a combined effect of mechanochemistry may play a more important role. The input of kinetic energy generated by mechanical stress, in combination with an oxidative environment, could have a greater impact on the molecular chain degradation.^27^ The input of kinetic energy into implanted mesh structures has the potential to create a lower activation energy barrier for the oxidative degradation processes. Not only by direct mechanical stress on molecular chains, but possibly also in conjunction with thermal conductivity,^49^, ^50^ both of which may have the ability to lower the activation energy required for polymer degradation.

## 4 CONCLUSIONS

To the authors' knowledge, this is the first study in which oxidation and surface fragmentation/degradation have been detected in explanted Ti‐PP mesh. The results obtained are similar to those of studies on explanted non‐coated PP mesh. Considering the data available, it is proposed that the Ti coating has an unquantified short‐term effect on reducing the rate of surface and bulk PP oxidation. This reduction is notable when comparing short‐ and long‐term explanted Ti‐PP meshes. However, as this study shows, over time environmental stress cracking still persists even with the initial full Ti coating, resulting in the removal of the coating and in the exposure of PP, creating a potential route for mesh oxidation. Our findings therefore suggest that oxidation is to be expected during the lifelong implantation of a Ti‐PP mesh in a similar pattern to that observed on uncoated PP mesh. These findings emphasize the need for future studies to gain a deeper understanding of both the characteristics of the implantation materials and the implantation environment in order to develop materials that can better withstand both the oxidative and mechanical stresses of lifelong implantation.

## CONFLICT OF INTEREST

The authors declare no conflicts of interest.

## Supporting information


**Data S1:** Supporting Information

## Data Availability

Data available on request from the authors.
