# Supplementary material for: Characterization in respect to degradation of titanium‐coated polypropylene surgical mesh explanted from humans
Source: J Biomed Mater Res B Appl Biomater. 2023 Jan 7;111(5):1142–52. doi: 10.1002/jbm.b.35221 (PMC10952695; doi:10.1002/jbm.b.35221)
Supplement: Supplementary file 1 — Data S1: Supporting Information [file JBM-111-1142-s001.docx]

# Supporting Information

# Characterisation in respect to degradation of titanium-coated polypropylene surgical mesh explanted from humans.

N. T. H. Farr^1,2^, B. Klosterhalfen^3^, G. K. Noé^4^

^1^ Department of Materials Science and Engineering, University of Sheffield, Sheffield, UK

^2^ Insigneo Institute for in silico Medicine, Sheffield, UK

^3^ Institute for Pathology at the Düren Hospital, Düren, Germany

^4^ University of Witten Herdecke, Department of Obstetrics and Gynecology Rheinlandclinics Dormagen, Dormagen, Germany

**SI-Fig.1:** Images **[A]** and **[B]** show electron images of the short-term (ST) and long-term (LT) implanted Ti-PP meshes, respectively, with 5 regions each from which EDS spectra were collected. **[C]** and **[B]** respectively show the EDS spectrum 1 for the two meshes.

**
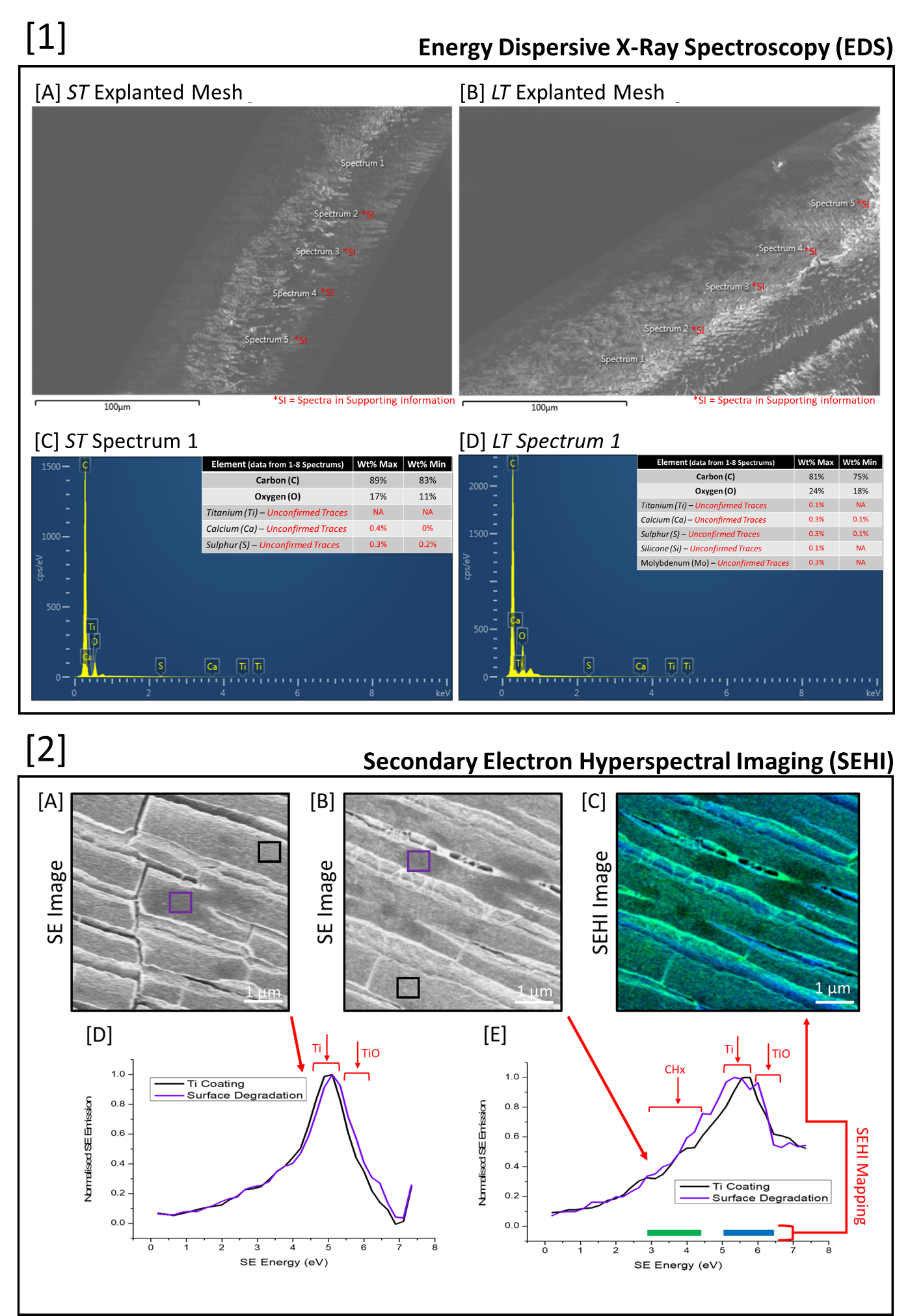
**

EDS results are presented in SI-Fig. 1. Five EDS spectra were collected of both the short-term (ST) and long-term (LT) implanted Ti-PP mesh. SI-Fig. 1C & 1D each show an example of an EDS spectra of the meshes. EDS was selected as an analytical method in accordance with the recommendation of ISO 10993-18:2020 for the identification of metals/alloys in medical devices. In this instance, EDS was performed with the aim of identifying the Ti coating on the explanted meshes. For both meshes, EDS was unable to confirm the presence of Ti. Traces of Ti, Calcium (Ca), Sulfur (S), Silicone (Si) and Molybdenum (MO) were identified but could not be confirmed via the EDS elemental detection software. It is most likely that these traces are identified as a result of a data processing error. In terms of the Ti coating, which is expected to be about 30 nm thick, it is expected that the identification using EDS is difficult because X-rays are generated in a region of about 2 µm in depth.

The only two elements that could be confirmed using EDS on both explanted meshes were carbon (C) and oxygen (O). Comparing the C/O ratio of the two Ti-PP meshes, it is seen that a trend of a greater percentage of oxygen is notable in the LT mesh. This result is comparable to that of FTIR previously presented. However, EDS is not suited to the analysis of low atomic number elements, and readings of C/O data acquired from EDS are not widely accepted, despite studies having previously published C/O ratios [53]. The authors would not recommend applying much academic significance to these results apart from stating that both meshes are highly likely to contain both carbon and oxygen. Since EDS does not provide the necessary surface sensitivity to assess a surface coating, SEHI was additionally performed.
